# The relationship between social support and employability among university students: a chain mediation model of psychological capital and career adaptability

**DOI:** 10.3389/fpsyg.2026.1848612

**Published:** 2026-08-31

**Authors:** Huagang Yang, Minglei Yue, Mingfei Jin, Yuan Tian, Wei Zhao

**Affiliations:** 1 School of Educational Sciences, Liaocheng University, Liaocheng, China; 2 Liaocheng University Dongchang College, Liaocheng, China; 3 School of Psychology, Zhejiang Normal University, Jinhua, China; 4 School of International Education, Taishan University, Taian, China

**Keywords:** career adaptability, employability, psychological capital, social support, university students

## Abstract

**Introduction:**

To further examine the association between social support and employability among university students and to verify the sequential mediation of psychological capital and career adaptability in this process, the present study administered the Social Support Rating Scale, the Positive Psychological Capital Questionnaire (PPQ), the Career Adapt-Abilities Scale (CAAS), and the College Students' Employability Self-Assessment Scale to 1765 university students.

**Methods:**

A total of 1,765 university students completed the self-report questionnaires measuring social support, psychological capital, career adaptability, and employability.

**Results:**

The results revealed the following: (1) social support, psychological capital, career adaptability, and employability were all significantly and positively correlated with one another (*p* < 0.01); (2) Psychological capital and career adaptability played a partial mediating role in the relationship between social support and employability. The unstandardized mediating effect values of the two independent mediation paths were 0.662 and 0.360, respectively, Psychological capital and career adaptability also exerted a significant chain mediating effect on the above relationship, with an unstandardized chain mediating effect value of 0.161.

**Discussion:**

These findings highlight psychological capital and career adaptability as key mediating mechanisms through which social support fosters employability among university students.

## 1 Introduction

In recent years, as higher education has become increasingly accessible and competition in the job market has intensified, university students have been facing mounting employment pressure. Statistics indicate that the number of graduates from regular higher education institutions in China is expected to reach 12.70 million in 2026, an increase of 480,000 compared with the 2025 cohort, further intensifying labor market competition. Against this backdrop, identifying the factors that influence university students' employability has become a central concern in academic research. Among these factors, social support, psychological capital, and career adaptability have been recognized as critical determinants of individuals' employment outcomes. Our research aims to investigate how social support influences employability through the chain mediating roles of psychological capital and career adaptability. By elucidating this mediating mechanism, the research not only provides more targeted guidance for enhancing students' employability but also offers empirical evidence to support mental health education and career development initiatives in higher education institutions.

### 1.1 Social support and employability

Social support is defined as the emotional, material, informational, and evaluative resources an individual receives within their social network, exerting a profound influence on psychological wellbeing and behavioral outcomes. For university students, sources of social support include family, friends, educational institutions, and peer groups. Social support not only provides emotional comfort but also offers information, resources, and guidance that enable students to better cope with challenges encountered during the job-seeking process. Within research on employability, social support has been identified as a crucial determinant. Prior studies have shown that social support enhances students' self-efficacy, thereby strengthening their confidence and motivation when facing employment challenges (Cohen and Wills, 1985). Moreover, it facilitates access to employment-related information and resources, aiding students in more effectively engaging in career planning and job-search activities (Kao et al., 2022). Nevertheless, the direct effects of social support on employability and the underlying mechanisms through which this influence occurs remain to be further explored.

### 1.2 The structure and influence of psychological capital

The concept of psychological capital first emerged within the field of human resource management. Drawing primarily on Luthans and Youssef (2004) framework, psychological capital is defined as a positive psychological state that individuals exhibit throughout their growth and development. This construct transcends human and social capital, representing a core psychological resource that plays a pivotal role in personal development and performance enhancement. Psychological capital comprises four dimensions—self-efficacy, hope, optimism, and resilience. Specifically, self-efficacy reflects an individual's strong belief in their capacity to accomplish specific tasks; hope involves goal setting and the identification of pathways to achieve those goals; resilience refers to the ability to recover and adapt effectively in the face of adversity; and optimism denotes a positive expectation of future outcomes. Psychological capital has been identified as a crucial determinant of employability, as it enhances positive emotions, mitigates negative affect, and strengthens individuals' capacity to cope with employment challenges (Avey et al., 2011). Nevertheless, the interactive mechanism through which psychological capital and social support jointly influence employability remains an important topic warranting further investigation.

### 1.3 Career adaptability and employability

Career adaptability refers to an individual's capacity to cope with uncertainty and change during the process of career development. It encompasses multiple dimensions, including self-awareness, environmental awareness, decision-making ability, and career planning skills (Savickas, 2005). As a key determinant of successful employment among university students, career adaptability enables individuals to gain a deeper understanding of their interests, abilities, and values, as well as the demands and opportunities of the external labor market (Zikic and Klehe, 2006). Empirical research has demonstrated a positive link between career adaptability and employability (Jia et al., 2024); however, the specific mechanisms through which social support influences employability via career adaptability remain insufficiently understood.

### 1.4 Theoretical basis of the chain mediation effect

**Figure 1:**
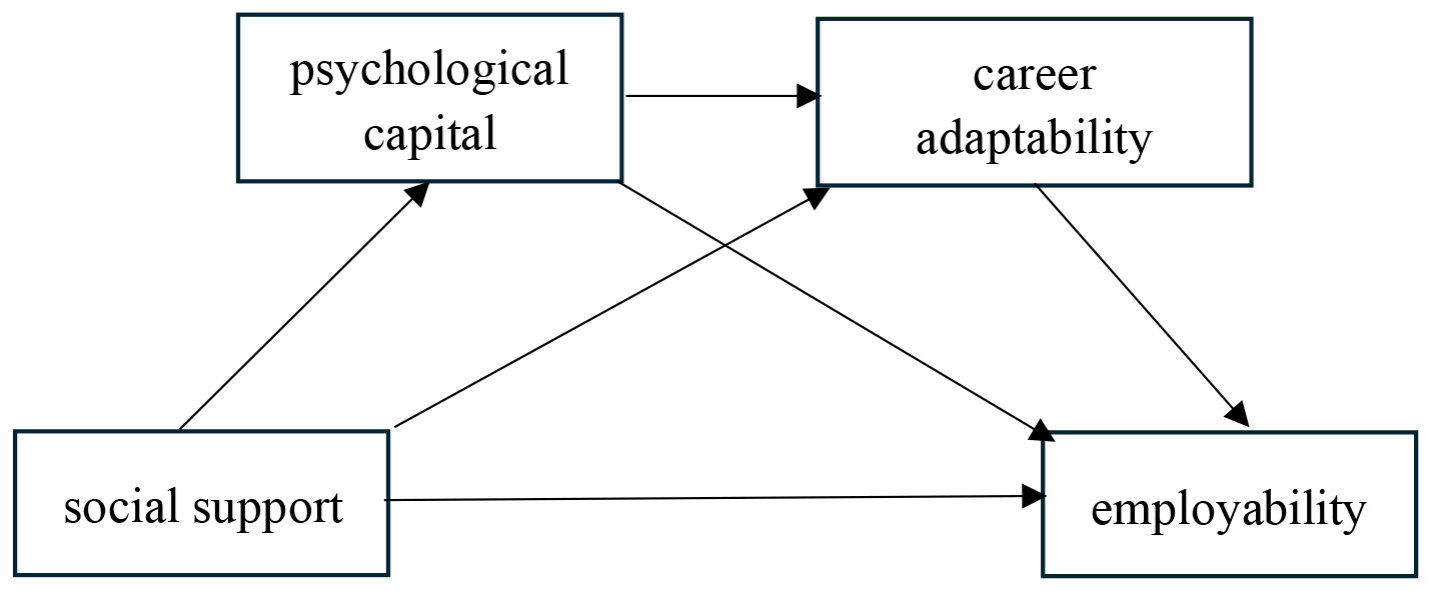
Hypothesized chain mediation model of psychological capital and career adaptability.

The chain mediation effect refers to a phenomenon in which one variable indirectly influences another through two or more sequential mediating variables (Hayes, 2013). In the present study, we hypothesize that psychological capital and career adaptability may jointly constitute a chain mediating pathway through which social support affects employability (see Figure 1). This theoretical framework is grounded in social cognitive theory, which emphasizes that an individual's social environment, such as social support, can shape their psychological states, including psychological capital, and behavioral strategies, such as career adaptability, thereby influencing employability. Nevertheless, the application and empirical validation of this framework in the field of university student employability remain limited and warrant further investigation.

## 2 Methods

### 2.1 Participants

The present research recruited college students from a higher education institution in Shandong Province as participants, distributing a total of 2,571 questionnaires. After removing extreme cases with completion times below or above three standard deviations from the mean, 1,765 valid questionnaires were retained, yielding an effective response rate of 68.7%. Among the respondents, 516 were male, accounting for 29.2% of the sample, and 1,249 were female, representing 70.8%. In terms of grade distribution, 1,150 first-year students comprised the largest proportion at 65.2%, followed by 304 second-year students (17.2%), 136 third-year students (7.7%), and 175 fourth-year students (9.9%).

### 2.2 Measures

The study employed four scales designed to assess social support, psychological capital, career adaptability, and employability.

#### 2.2.1 Social support rating scale

Social support was measured with the Social Support Rating Scale proposed by Xiao (1994). This scale consists of 13 items, including 11 single-choice questions and two multiple-choice questions. Single-choice items are rated on a four-point scale, while scores for multiple-choice items are determined by the number of options selected. The overall score is derived from the sum of all item scores. Regarding dimensional structure, items 1, 3, 4, 5, 6, 7, and 8 primarily assess perceived (subjective) support; items 2, 9, and 10 measure objective support; and items 11, 12, and 13 evaluate the extent to which individuals utilize available support. In the present study, the Cronbach's α coefficient of the Social Support Rating Scale was 0.840. Confirmatory factor analysis demonstrated that the average variance extracted (AVE) values of the three dimensions ranged from 0.508 to 0.598 (all higher than 0.5), and composite reliability (CR) values were between 0.775 and 0.815 (all exceeding 0.7). These findings confirmed favorable convergent validity of the research data, which fully met the relevant evaluation criteria.

#### 2.2.2 Positive psychological capital questionnaire (PPQ)

The present research employed the positive psychological capital questionnaire developed by Zhang et al. (2010) to assess psychological capital. The questionnaire is composed of four dimensions: self-efficacy (items 1, 3, 5, 7, 9, 11, 13), resilience (items 2, 4, 6, 8, 10, 12, 14), hope (items 15, 17, 19, 21, 23, 25), and optimism (items 16, 18, 20, 22, 24, 26), totaling 26 items. All items are recorded on a seven-point Likert scale. Higher scores indicate greater psychological capital. In this study, the Cronbach's α coefficient of the questionnaire was 0.938. Confirmatory factor analysis showed that the average variance extracted (AVE) values of the four factors ranged from 0.571 to 0.727, all exceeding 0.5, while composite reliability (CR) values were between 0.767 and 0.949, all above 0.7. These results demonstrated satisfactory convergent validity of the data used in this analysis, and all indicators met the evaluation criteria.

#### 2.2.3 Career adaptability scale

The Career Adaptability Scale, proposed by Wu (2008) and based on Savickas (2005) theoretical framework, was used to measure career adaptability. The scale comprises 21 items across four dimensions, each of which corresponds to career confidence, career curiosity, career concern, and career control. Specifically, the career curiosity dimension includes items 5, 7, 8, 12, 15, and 18 (six items); career control includes items 1, 10, 13, and 19 (four items); career confidence includes items 2, 4, 6, 9, 14, 16, and 17 (seven items); and career concern includes items 3, 11, 20, and 21 (four items). Items employ a five-point Likert scale for rating. Items 4, 10, 11, and 13 are reverse-scored, with scores ranging from one (“strongly agree”) to five (“strongly disagree”). In the present study, the Cronbach's α coefficient of the scale was 0.914. Confirmatory factor analysis indicated that the average variance extracted (AVE) values of the four factors ranged from 0.536 to 0.569 and were all greater than 0.5, while composite reliability (CR) values were between 0.710 and 0.887, all higher than 0.7. The above results verified good convergent validity of the analyzed data, and all indicators met the relevant evaluation criteria.

#### 2.2.4 Employability scale

The Employability Scale, compiled by Lyu (2012), was used to assess university students' employability. A total of 26 items constitute the scale, with responses recorded on a five-point Likert scale. In terms of dimensional structure, self-development ability (i.e., self-innovation and challenge) includes items one to seven (seven items); interpersonal communication skills comprise items 8 to 12 (five items); employment confidence (i.e., confidence in employment and career development) includes items 13 to 17 (five items); practical ability (i.e., the integration of theory and practice) covers items 18 to 22 (five items); and adaptability (i.e., adaptive development) consists of items 23 to 26 (four items). Items 5, 18, and 23 are reverse-scored. The total score is obtained by summing all item scores. In the present study, the Cronbach's α coefficient of the scale was 0.954. Confirmatory factor analysis revealed that the average variance extracted (AVE) values of the five factors ranged from 0.555 to 0.736, all exceeding 0.5, and composite reliability (CR) values were between 0.813 and 0.933, all above 0.7. These results indicated favorable convergent validity of the analyzed data, and all indicators met the relevant evaluation criteria.

### 2.3 Data collection and processing

Data were collected through the “Wenjuanxing” online platform, ensuring anonymity and confidentiality. After all questionnaires were retrieved, invalid responses and outliers were excluded. Data analyses were carried out using SPSS 25.0 in conjunction with the PROCESS macro (version 4.0, Model 6). Descriptive statistics, correlation analysis, and regression analysis were employed to examine the data.

## 3 Results

### 3.1 Common method bias test

Harman's single-factor test was adopted to examine common method bias. An unrotated exploratory factor analysis was conducted on all items of the scales. The results showed that the explanatory variance rate of the first common factor was 43.00%, slightly higher than the reference threshold of 40%. It indicated that a certain degree of common method bias existed in this study, which was still within an acceptable range.

### 3.2 Correlation analysis

Pearson's correlation method was applied to analyze the associations among social support, psychological capital, career adaptability, and employability. As displayed in Table 1, all variables were significantly positively correlated, with the strongest correlation observed between psychological capital and career adaptability. Specifically, social support showed a significant positive relationship with psychological capital (*r* = 0.584, *p* < 0.01), career adaptability (*r* = 0.576, *p* < 0.01), and employability (*r* = 0.572, *p* < 0.01). Psychological capital was also significantly positively correlated with career adaptability (*r* = 0.785, *p* < 0.01) and employability (*r* = 0.849, *p* < 0.01). Similarly, career adaptability was significantly positively correlated with employability (*r* = 0.826, *p* < 0.01).

**Table 1:** Correlations among variables (*n* = 1,765).

Variable	1	2	3	4
1. Social support	1			
2. Psychological capital	0.584^**^	1		
3. Career adaptability	0.576^**^	0.785^**^	1	
4. Employability	0.572^**^	0.849^**^	0.826^**^	1

* *P* < 0.05.

** *P* < 0.01.

*** *P* < 0.001.

### 3.3 Chain mediation effect analysis

**Figure 2:**
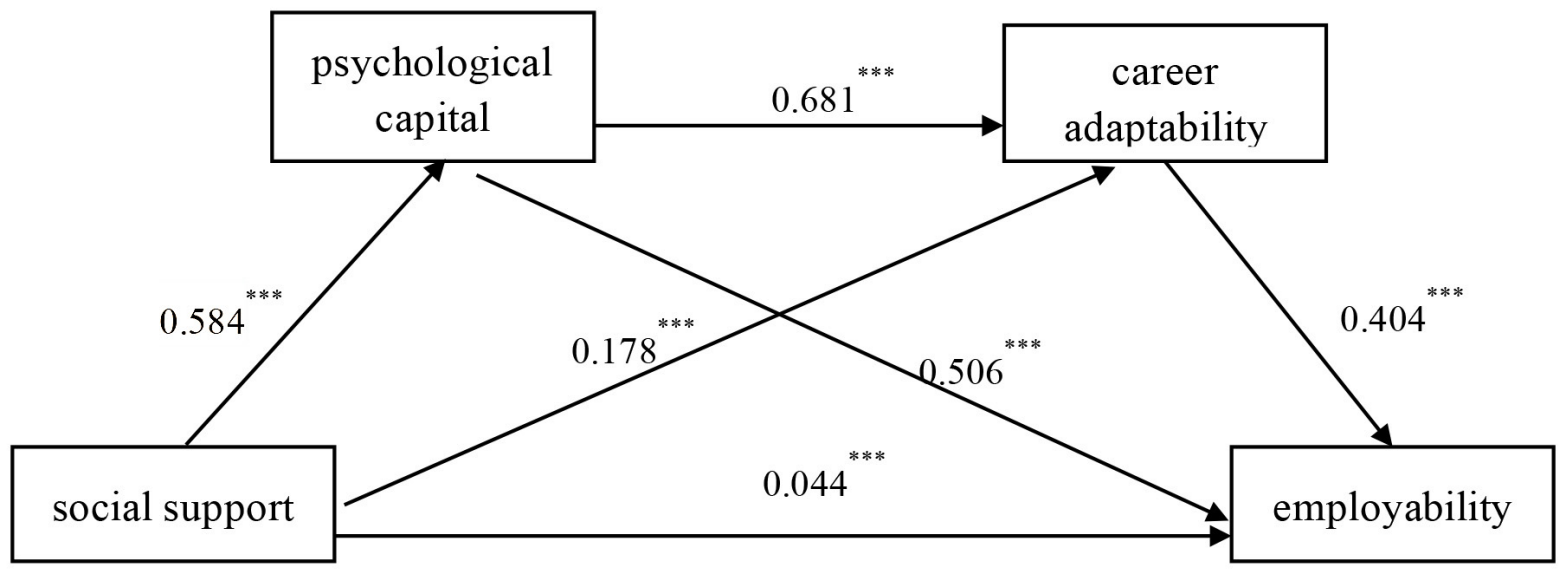
Path diagram of the influence exerted by social support on employability. ^***^P < 0.001.

As shown in Table 2, social support significantly positively predicted psychological capital (β = 0.584, *p* < 0.001). Both social support (β = 0.178, *p* < 0.001) and psychological capital (β = 0.681, *p* < 0.001) were significant positive predictors of career adaptability. Moreover, psychological capital significantly predicted employability (β = 0.506, *p* < 0.001). Based on these results, the pathway through which social support influences employability is illustrated in Figure 2.

**Table 2:** Regression equations among variables (*n* = 1,765).

Dependent variable	Predictor	*R*	*R2*	*F*	β	*t*
Psychological capital		0.584	0.341	913.301		
	Social support				0.584	30.221^***^
Career adaptability		0.798	0.637	1543.892		
	Social support				0.178	10.047^***^
	Psychological capital				0.681	38.488^***^
Employability		0.888	0.789	2,190.881		
	Social support				0.044	3.193^***^
	Psychological capital				0.506	27.632^***^
	Career adaptability				0.404	22.226^***^

^***^P < 0.001.

Specifically, psychological capital significantly and positively influenced career adaptability (β = 0.681, *t* = 38.488, *p* < 0.001), indicating that psychological capital plays a crucial role in helping individuals adapt to career-related challenges and changes. Individuals with higher levels of psychological capital are more likely to demonstrate stronger career adaptability, which may be attributed to their greater self-efficacy, hope, optimism, and resilience. Social support was also found to significantly and positively influence career adaptability (β = 0.178, *t* = 10.047, *p* < 0.001), suggesting that support derived from one's social network—such as emotional, informational, and material support—can substantially enhance career adaptability. In the regression model predicting employability, both psychological capital (β = 0.506, *t* = 27.632, *p* < 0.001) and career adaptability (β = 0.404, *t* = 22.226, *p* < 0.001) exhibited significant positive effects, underscoring their importance as key factors promoting employability. The direct effect of social support on employability was significant (β = 0.044, *t* = 3.193, *p* < 0.01), which indicates that social support plays an important role in the formation of individual employability.

To further examine the association between social support and employability, the bias-corrected bootstrap method was applied for testing. As shown in Figure 2, psychological capital and career adaptability played partial mediating roles linking social support to employability. The mediation analysis revealed that the indirect effects of psychological capital and career adaptability were highly significant, with a total indirect effect value of 1.183. This finding indicates that social support primarily influences employability indirectly through these two mediators. Moreover, this effect was significant within the 95% confidence interval (1.103–1.264), further confirming the statistical importance of the mediating effect.

Specifically, the mediating effects of psychological capital and career adaptability in the relationship between social support and employability include three paths. The first path is social support → psychological capital → employability [effect = 0.662, 95% CI (0.588, 0.737)]. Since the 95% confidence interval of the effect does not include zero, the mediating effect of psychological capital is significant, indicating that social support can enhance employability by increasing individuals' psychological capital. The second path is social support → career adaptability → employability [effect = 0.161, 95% CI (0.124, 0.200)]. Similarly, the 95% CI excludes zero, showing a significant mediating role of career adaptability; by improving individuals' career adaptability, social support can indirectly promote employability. The third path is social support → psychological capital → career adaptability → employability [effect = 0.360, 95% CI (0.305, 0.419)]. The 95% CI of this chained mediation also does not include zero, providing evidence for a significant serial mediation involving psychological capital and career adaptability (see Table 3). These results suggest that psychological capital and career adaptability play important mediating roles in the relationship between social support and employability. In other words, social support not only directly influences psychological capital but also further enhances career adaptability through psychological capital, ultimately promoting the improvement of employability.

**Table 3:** Mediation analysis of psychological capital and career adaptability between social support and employability (*n* = 1,765).

Path / Effect	Effect value	Boot SE	95% CI lower	95% CI upper	Relative indirect effect
Total indirect effect	1.183	0.041	1.103	1.264	0.922
Ind1 social support → psychological capital → employability	0.662	0.038	0.588	0.737	0.516
Ind2 social support → career adaptability → employability	0.161	0.019	0.124	0.200	0.125
Ind3 social support → psychological capital → career adaptability → employability	0.360	0.029	0.305	0.419	0.281
Ind1–Ind2	0.502	0.049	0.405	0.597	–
Ind1–Ind3	0.302	0.054	0.198	0.408	–
Ind2–Ind3	−0.199	0.031	−0.263	−0.140	–

## 4 Discussion

### 4.1 Associations of social support with university students' employability

The results of this study indicate a significant positive correlation between social support and college students' employability, which is consistent with previous findings (Cohen and Wills, 1985; Chen, 2023; He and Yao, 2018). Social support provides college students with emotional comfort and material assistance, helping them build confidence, which is positively correlated with their ability to cope with employment challenges. In addition, social support can offer valuable employment information and resources. Such support is positively linked to students' career development as well as their access to job opportunities.

### 4.2 The mediating role of psychological capital

The results of this study indicate that psychological capital exerts a mediating effect between social support and employability, which is consistent with the findings of Chen (2023) and Liu (2022). Specifically, individuals with higher psychological capital tend to establish clearer career goals, participate more actively in job-hunting activities, and exhibit stronger adaptability and frustration resilience when confronted with employment difficulties.

### 4.3 The mediating role of career adaptability

Career adaptability also serves as a mediator between social support and employability. Social support is positively associated with individuals' career adaptability, helping them better plan their careers and make more rational career decisions, so as to improve employability.

### 4.4 Chain mediating effect

Our results highlight the mediating roles of psychological capital and career adaptability between social support and employability. Notably, psychological capital demonstrated a significant mediating effect, which aligns with previous research emphasizing its importance for individual career development. Additionally, career adaptability also served as a mediator between social support and employability, indicating that individuals' capacity to adapt to career changes is a key factor in enhancing employability.

In conclusion, this study reveals that social support indirectly influences employability through psychological capital and career adaptability, offering significant theoretical support and practical guidance for career development and human resource management. Future research could further investigate the relationships among these variables across diverse cultural and social contexts and explore strategies for designing effective interventions to enhance individuals' psychological capital and social support, thereby fostering improvements in employability.

### 4.5 Theoretical and practical implications

The present study holds important theoretical and practical value in its contribution to enriching the theoretical framework regarding the impact of social support, psychological capital, and career adaptability on employability. It also provides empirical evidence for how universities can enhance students' employability by offering social support and fostering psychological capital and career adaptability. Furthermore, the findings suggest that higher education institutions should emphasize the cultivation of psychological capital and career adaptability in career guidance services to promote students' holistic development and successful employment outcomes.

### 4.6 Limitations and future research directions

Although this study provides valuable insights, several limitations should be acknowledged. First, the cross-sectional research design precludes the determination of causal relationships among the variables. Future studies could employ longitudinal designs to better understand the dynamic relationships among these variables. Second, the sample was primarily drawn from a single region in Shandong Province, which may somewhat limit the generalizability of the findings. Future research could consider samples from other provinces or cross-cultural contexts to test the model's broader applicability. Finally, this study focused on the overall effect of social support; future research could further examine the specific impacts of different types of social support, such as support from family, friends, or the university, on employability.

## 5 Conclusion

### 5.1 Key findings

This study yielded the following key findings based on empirical analysis:

1. Direct effect of social support: social support plays a significant positive role in shaping college students' employability, indicating that it is a crucial factor for successful employment.
2. Mediating role of psychological capital: psychological capital partially mediates the relationship between social support and employability, highlighting its role as an important psychological mechanism linking social support to employability.
3. Mediating role of career adaptability: career adaptability also serves as a mediator between social support and employability, revealing its essential role in enhancing college students' employability.
4. Chain mediating effect: psychological capital and career adaptability jointly form a chain mediating effect linking social support and employability, providing a new perspective for understanding how social support indirectly influences employability.

### 5.2 Recommendations for university career guidance

Based on the findings of this study, the following recommendations are proposed:

1. Strengthen social support networks: universities should focus on building and enhancing students' social support networks, including family, friends, teachers, and alumni, to provide greater emotional, informational, and resource support.
2. Foster psychological capital: universities can help students develop and enhance their psychological capital—such as self-efficacy, hope, resilience, and optimism—through mental health education, career planning guidance, and related programs.
3. Enhance career adaptability: higher education institutions should emphasize career education and guidance, assisting students in improving self-awareness, environmental awareness, decision-making, and planning abilities to better adapt to the demands of career development.

### 5.3 Recommendations for mental health education

This study highlights the importance of mental health in college students' career preparedness and thus proposes the following recommendations:

1. Integrate mental health education with career guidance: universities can combine mental health education with career guidance to enhance students' employability through improved psychological wellbeing.
2. Implement targeted mental health activities: institutions can organize mental health activities tailored to different academic years and disciplines to address the diverse needs of student groups.
3. Provide personalized and targeted psychological support: universities should conduct in-depth assessments to understand students' actual needs and offer individualized, targeted mental health services to help them address potential psychological challenges during career preparation.

## Data Availability

The raw data supporting the conclusions of this article will be made available by the authors, without undue reservation.
